# The biomechanical effect of the relevant segments after facet-disectomy in different diameters under posterior lumbar percutaneous endoscopes: a three-dimensional finite element analysis

**DOI:** 10.1186/s13018-021-02733-7

**Published:** 2021-10-14

**Authors:** Yin Shi, Yi-Zhou Xie, Qun Zhou, Yang Yu, Xiao-Hong Fan

**Affiliations:** 1 Hospital of Chengdu University of Traditional Chinese Medicine, No. 39 Shi-er-qiao Road, Chengdu, 610072 Sichuan Province People’s Republic of China; 2 Chengdu University of Traditional Chinese Medicine, No. 1166 Liu-tai Avenue, Chengdu, 611137 Sichuan Province People’s Republic of China

**Keywords:** Posterior lumbar percutaneous endoscopy, Facetectomy, Biomechanical effect, Three-dimensional finite element analysis

## Abstract

**Objective:**

To evaluate the biomechanical influence after percutaneous endoscopic lumbar facetectomy in different diameters on segmental range of motion (ROM) and intradiscal pressure (IDP) of the relevant segments by establishing three dimensional finite element (FE) model.

**Methods:**

An intact L3–5 model was successfully constructed from the CT of a healthy volunteer as Model A (MA). The Model B (MB), Model C (MC) and Model D (MD) were obtained through facetectomy on L4 inferior facet in diameters 7.5 mm, 10 mm and 15 mm on MA for simulation. The ROM and IDP of L3/4 and L4/5 of four models were all compared in forward flexion, backward extension, left and right bending, left and right rotation.

**Results:**

Compared with MA, the ROM of L4/5 of MB, MC and MD all increased. MD changed more significantly than MB and MC in backward extension, right bending and right rotation. But that of MB and MC on L3/4 had no prominent change, while MD had a slight increase in backward extension. The IDP of MB and MC on L4/5 in six states was similar to MA, yet MD increased obviously in backward extension, right bending, left and right rotation. The IDP on L3/4 of MB and MC was resemble to MA in six conditions, nevertheless MD increased slightly only in backward extension.

**Conclusion:**

Compared with the facetectomy in diameters 7.5 mm and 10 mm, the mechanical effect brought by facetectomy in diameter 15 mm on the operating segment changed more significantly, and had a corresponding effect on the adjacent segments.

## Introduction

Lumbar disc herniation (LDH) and lumbar spinal stenosis (LSS) are common etiologies for spine surgery. Low back pain, radialgia and numbness of lower extremity are frequent clinical symptoms, which seriously affect the life quality of patients [1, 2]. With the progress of science and technology and the change of people's life style, the diseases tends to appear among younger population [3]. Although open microdiscetomy is considered to be the gold standard method [4], the need for minimally invasive techniques and the improvements in the use of optics and surgical instruments have led to the utilization of PEID/PELD(PELD, Percutaneous Endoscopic Lumbar Discectomy; PEID, Percutaneous Endoscopic Interlaminar Discectomy). PEID and PELD, by virtue of its transforaminal approach, have several advantages over traditional open operations such as smaller wound, less blood loss, shorter hospital stays and hospitalization expenses, more rapid recovery, lower complication rate and infection rate [5]. With the promotion of lumbar percutaneous endoscopic instruments and the improvement of the technical level of clinicians, its indications are also expanding.

According to different approaches, lumbar percutaneous endoscopy can be divided into Percutaneous endoscopic transforaminal discectomy (PETD) and Percutaneous endoscopic interlamina discectomy (PEID) [6]. Surgeons need to choose different approaches based on different indications. In dealing with types of prolapse, subaxillary of lumbar disc herniation as well as lumbar spinal stenosis in area 1 and 2, the PEID is more advantageous [7]. The lumbar interlaminar space gradually narrows from bottom segment to top segment and the L5/S1 interlaminar space is more toward the rear. Thus, the natural passage between the segmental laminae is wider. In that case, the working channel could directly enter the interlaminar space to attach the back of the ligamentum flavum. In the superior segments such as L3/4, L4/5, the positional relation between the lamina is imbricate, and the natural passage of the interlaminar space is obviously narrower than that of L5/S1 [8]. In order to put the working channel through the narrow interlaminar space broadening its surgical area and facilitating operation so as to enlarge lateral recess to achieve the purpose of complete decompression, it is often necessary to perform inferior facetectomy [9]. Many previous studies [10–12] have shown that excessive destruction of articular process will lead to the destruction of lumbar stability. Consequently, corresponding fixation plays an irreplaceable role to against it. Then here comes the question, what is the exactly effect of facetectomy in different diameters on the mechanical stability of lumbar spine? This is an issue that must be dealt with for bony decompression under endoscopy without the use of screw-rod system. In this study, three-dimensional finite element method was implemented to simulate facetectomy on L4 inferior articular process under lumbar posterior percutaneous endoscopic in different diameters (7.5 mm, 10 mm, 15 mm), hereby to study the changes of ROM and IDP of operating segment (L4/5) and adjacent segment (L3/4) after facetectomy in different diameters.

## Objects and methods

### General information

A 30-year-old male volunteer with height 175 cm and weight 65 kg was selected to notify the research content and sign the informed consent form before the start of the study, which was reviewed by the ethics committee of the affiliated Hospital of Chengdu University of traditional Chinese Medicine, affiliated to the author. This study has been also approved by the internal ethics committee of the organization to which the author belongs. L1-S2 related imaging data were obtained by lumbar X-ray, CT and MRI. Besides, the lumbar degenerative diseases and spinal deformities were excluded.

### Software and equipment

Equipment in this study included Siemens Somatom Sensation 64 row helical CT (supported by the department of radiology, Hospital of Chengdu University of Traditional Chinese Medicine). Mimics 16.0(professional medical image application software); Creo3.0 (surface design professional software); Geomagic Studio 12.0 (3D modeling reverse engineering software); and ANSYS15.0 (finite element analysis software). All the above experimental software was provided by the key laboratory of biomechanics of Southern Medical University.

### Research methods

#### Establishment of normal L3-5 3D finite element model

**Fig. 1:**
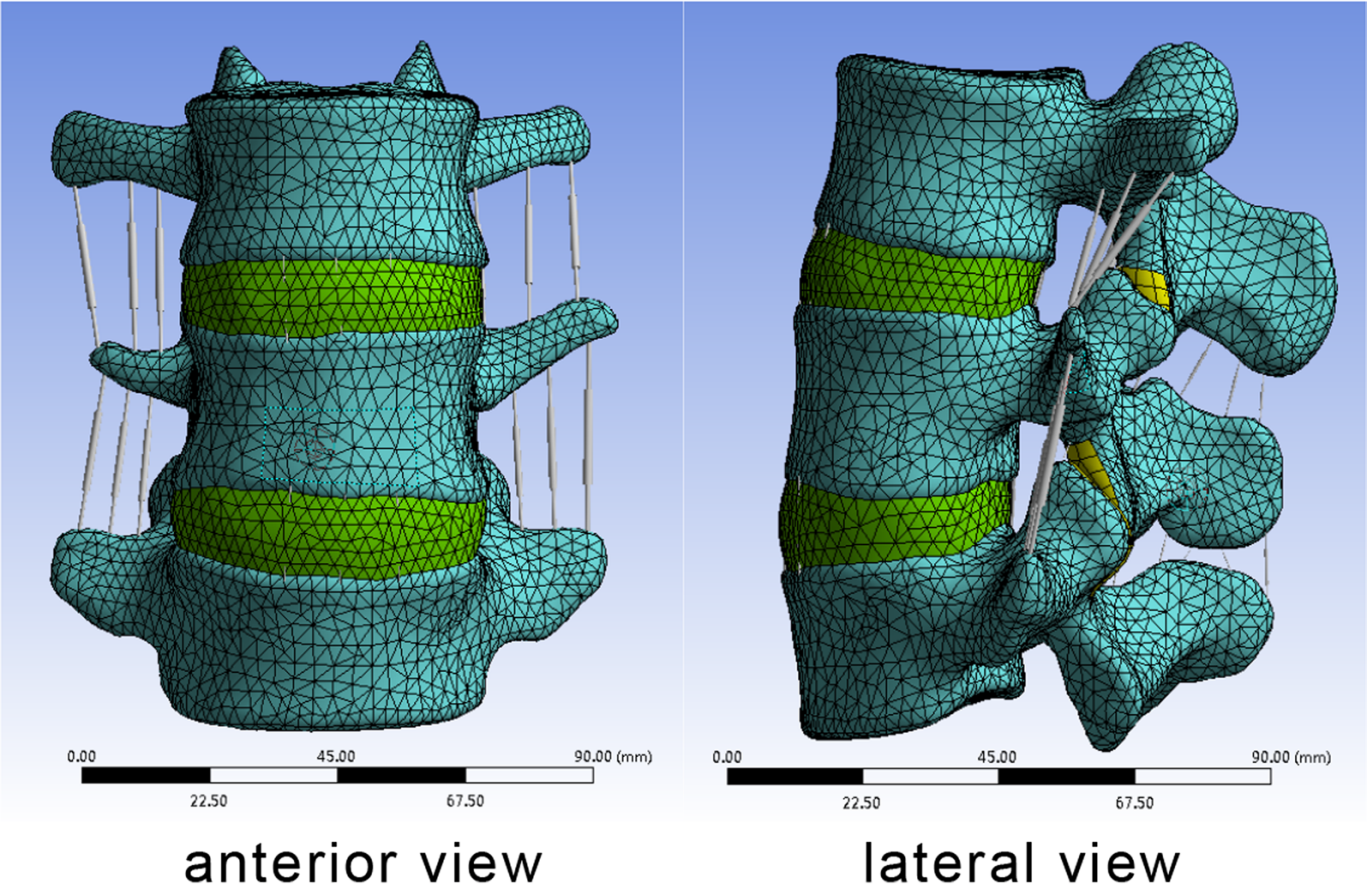
Normal three-dimensional finite element model of L3-5

The helical CT was used to scan the L1-S2, and the two-dimensional cross-sectional map of 0.625 mm was saved in DICOM format. Then the DICOM file was inputted into Mimics16.0 software, and the L3–5 three-dimensional model was established in Mimics software. After polishing and smoothing, the model was imported into ANSYS for meshing processing, and the bony finite element model was made. According to the anatomical position of each ligament, the normal three-dimensional finite element model of L3–5 was established by adding intervertebral disc, anterior longitudinal ligament, posterior longitudinal ligament, yellow ligament, interspinous ligament, supraspinous ligament and intertransverse ligament to the model which is shown in Fig. 1. The structure in the model is assigned according to the normal organization parameters which portrayed in Table 1 [13]. The starting and ending point and cross-sectional area refer to the anatomical observation of the relevant segments [14]. The articular surfaces of all joints in the model are defined as sliding contact relations, and the friction coefficient is 0.1 [15].

**Table 1:** material properties used to represent various components in the model

Items	Elasticity modulus (Mpa)	Passion ratio
Cortical bone	12,000	0.3
Cancellous bone	100	0.3
Cartilago articularis	25	0.4
Nucleus pulposus	1	0.49
Fibrous rings	4.2	0.45
Anterior longitudinal ligaments	7.8	0.30
Posterior longitudinal ligaments	10	0.30
Ligamentum flavum	15	0.30
Intertransverse ligaments	10	0.30
Capsule ligament	7.5	0.30
Interspinous ligaments	10	0.30
Supraspinal ligaments	8	0.30

The finite element model of L3–5 (M1) is established by scanning the lumbar of a 30-year-old young male volunteer through Siemens Somatom Sensation64 multi-sliced spiral CT (MSCT) and constructing with ANSYS and MIMICS software.

#### Verifying the validity of the model

The finite element model obtained in this study was compared and analyzed with various data obtained from autopsy, including SHIM [16]. Comparisons were conducted under the same environment, condition constraints and load, and with the full range of activities in all directions. Additionally, ligament data at each position were modified to ensure model data was within the range of biomechanical data obtained by anatomy such as SHIM [16], hence ensuring the effectiveness and reliability of modeling which could be seen in Table 2.

**Table 2:** Results from validation of the finite element model (°)

Items	Shim specimen test		L3–5 finite element	
	L3/4	L4/5	L3/4	L4/5
Torque	7.5		7.5	
flexion	4.2 ± 0.8	5.4 ± 0.9	3.9	4.6
extension	2.9 ± 0.5	2.9 ± 0.5	3.3	3.2
Left flexion	3.5 ± 1.0	4.4 ± 1.1	3.5	3.4
Right flexion	3.5 ± 1.0	4.4 ± 1.1	3.5	3.4
Left rotation	2.8 ± 0.6	3.8 ± 1.0	2.7	2.8
Right rotation	2.8 ± 0.6	3.8 ± 1.0	2.7	2.8

#### Establishment of facetectomy models in different diameters

**Fig. 2:**
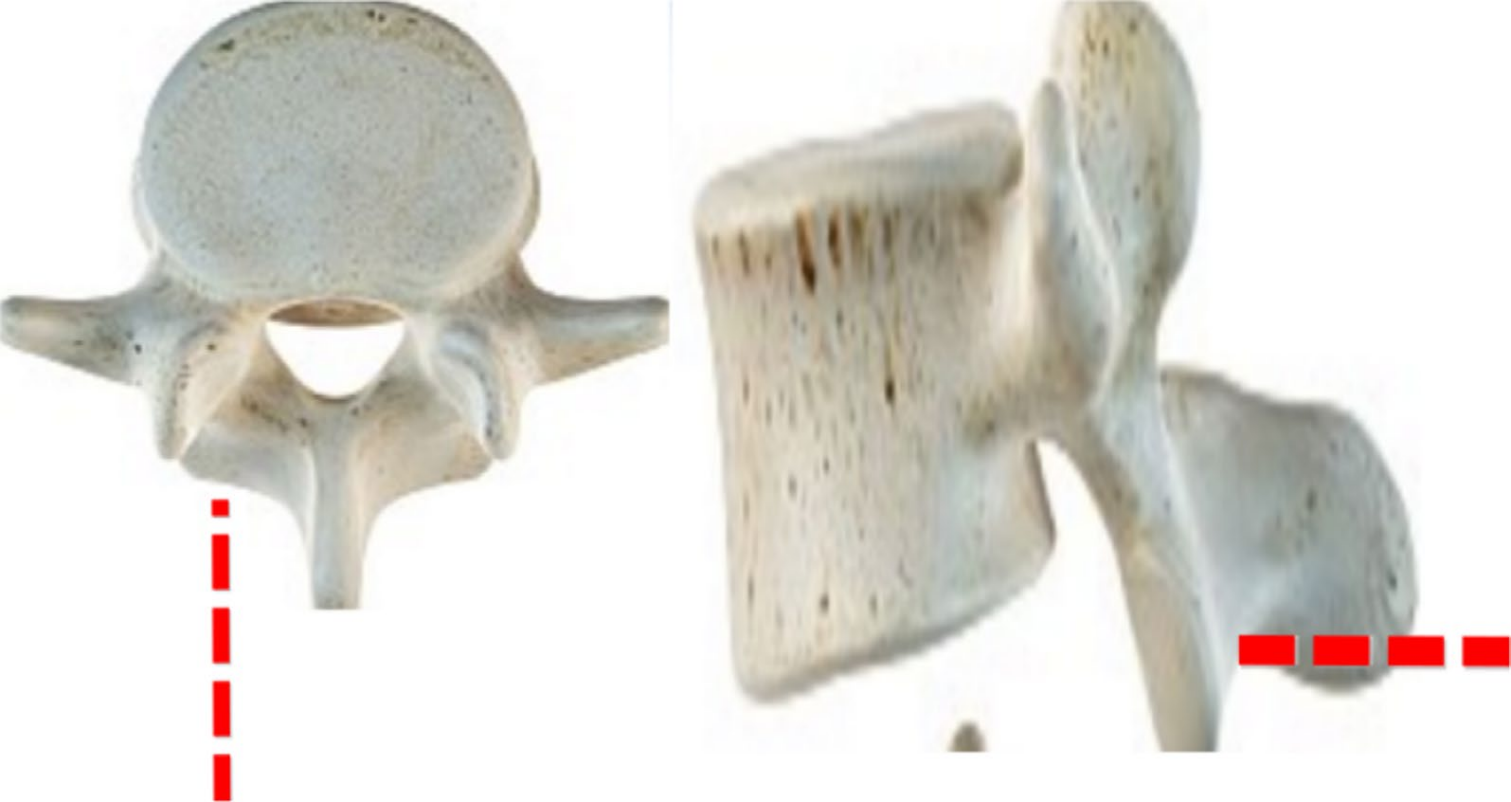
The path of facetectomy the left inferior articular process of L4 was regarded as the puncture target to simulate the vertical puncture route

**Fig. 3:**
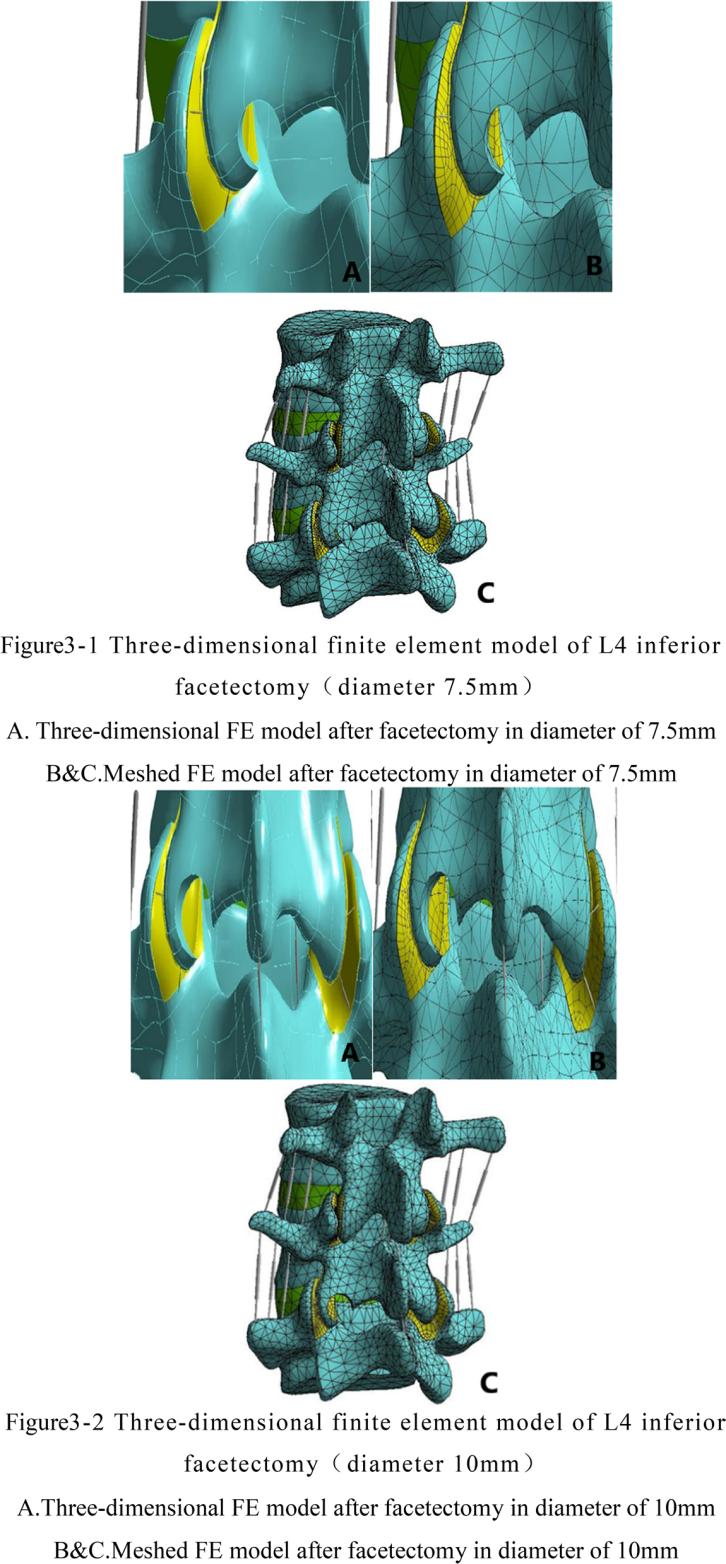

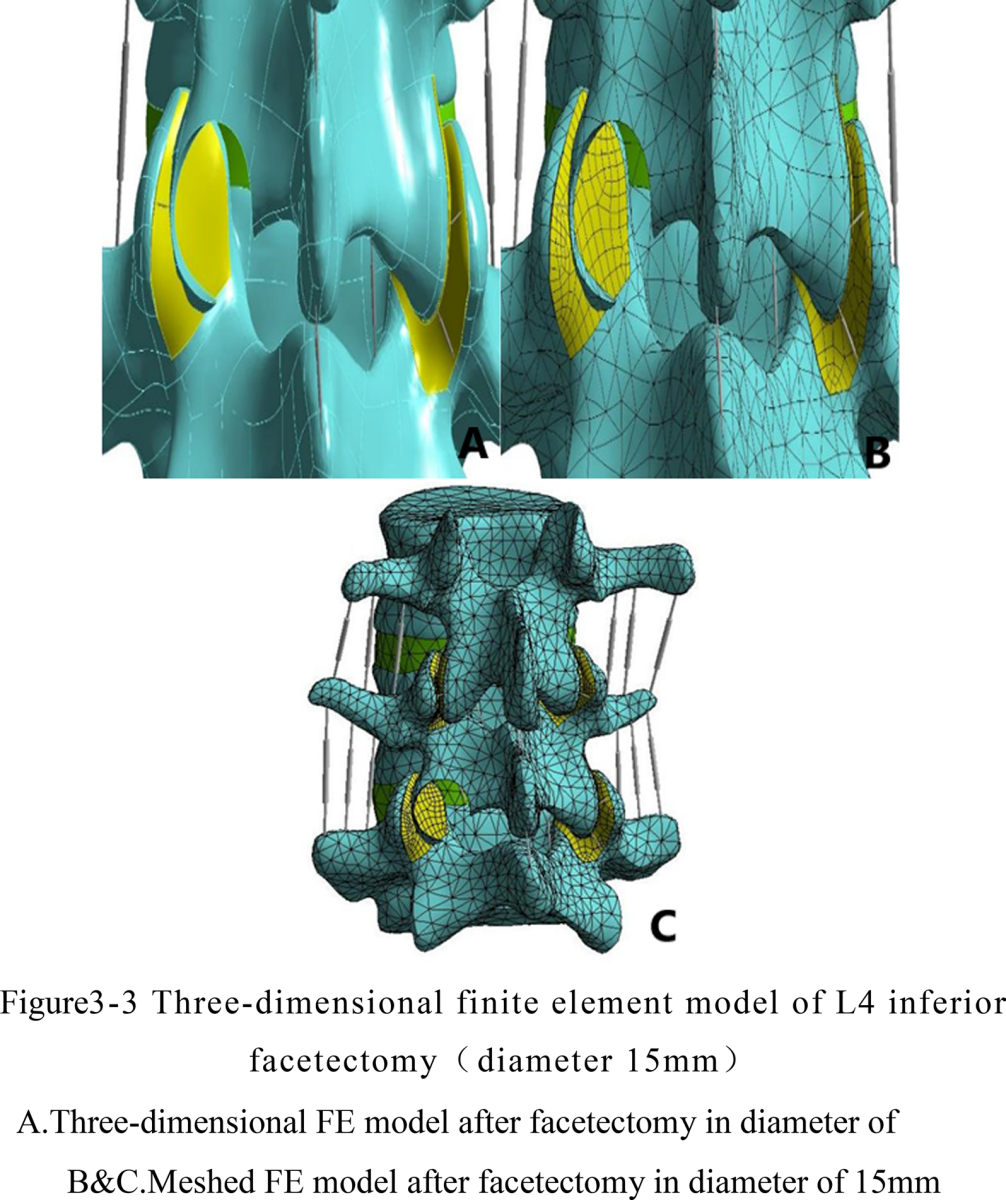
(1) Three-dimensional finite element model of L4 inferior facetectomy (diameter 7.5 mm). **a** Three-dimensional FE model after facetectomy in diameter of 7.5 mm. **b**, **c** Meshed FE model after facetectomy in diameter of 7.5 mm. (2) Three-dimensional finite element model of L4 inferior facetectomy (diameter 10 mm). **a** Three-dimensional FE model after facetectomy in diameter of 10 mm. **b**, **c** Meshed FE model after facetectomy in diameter of 10 mm. (3) Three-dimensional finite element model of L4 inferior facetectomy (diameter 15 mm). **a** Three-dimensional FE model after facetectomy in diameter of 15 mm **b**, **c** Meshed FE model after facetectomy in diameter of 15 mm

Based on the established L3–5 normal finite element model, the percutaneous endoscopic facetectomy with the posterior approach of L4 was simulated, and the left inferior articular process of L4 was used as the puncture target to simulate the vertical puncture route in clinical operation (Fig. 2). Before the disectomy, the articular capsule and capsule ligaments were resected as same as the operational process to reveal the facet joint.The reamers with diameters of 7.5 mm, 10 mm and 15 mm were selected to simulate the reamer. Then the cylindrical tools reached the L4 inferior articular process along the route. Firstly, we mark the site where the reamer coincides with the inferior articular process of L4 as the disectomy area of the articular process. Secondly, we dissect this part of the bone structure, dealing with the ligament structure destroyed on the path of reamer. Finally, we use the software to optimize the surface grid and body grid of the inferior articular process resection area of L4, and three-dimensional finite element models (model B/C/D) of facetectomy in diameter 7.5 mm, 10 mm and 15 mm were obtained respectively, as is clearly shown in see Fig. 3.

#### Boundary and load

In this research, the inferior surface of the L5 vertebral remained immobilized throughout the load simulation. The L3 segment was physiologically loaded with 400 N. Afterward, a bending moment of 7.5 Nm was applied to the L3 vertebra to recreate backward extension, forward flexion, left and right lateral bending, and left and right axial rotation. All loads were chosen according to Shim et al. [16].

#### Analysis of the biomechanical changes of the model before and after facetectomy

Based on the simulating the relevant procedures to the normal finite element model, the postoperative model was obtained. We acquired the influence cloud map of the discussion area to get the maximum Von Mises stress, extreme value through the finite element method. IAR was used to measure each segment of the model after processing, Range of ROM. The following standardized formula is used for calculation:

$${\text{NVs }} = ({\text{Vs}} - {\text{Vm)/ Vm }} \times { 1}00\%$$

$${\text{NRi }} = ({\text{Ri}} - {\text{Rm)/Rm }} \times { 1}00\%$$

Among them, NVs (Normalized Von Mises) and NRi (Normalized Rotation) are the standard deviation values of the difference between the post-processing model and the pre-processing model. Vs and Ri represent the maximum stress and activity range of the normal model after processing, and Vm and Rm represent the maximum stress and activity range of the normal model. The divergences in the biomechanical changes caused by the relevant operation to the normal model could be clearly clarified by NVs and NRi.

## Results

This part of the study established a validated effective L3–L5 three-dimensional finite element model of the lumbar spine, which simulated facetectomy in different diameters in the L4/5 lumbar percutaneous endoscopic surgery, and imitated the vertical puncture route in clinical operation aiming the inferior articular process of L4 as the target of facetectomy. Along the path, reamers with diameters of 7.5 mm, 10 mm, and 15 mm resected the L4 inferior articular process. Thus, the three-dimensional finite element models with disectomy diameters of 7.5 mm (MB), 10 mm (MC) and 15 mm (MD) on the L4 inferior articular process were obtained. The above models had good geometric shape and high simulation degree which is qualified to be used for corresponding biomechanical analysis. The normal three-dimensional finite element model of L3–L5 and the three models of which L4 articular processes with disectomy in different size were applied in the six directions with the same load as mentioned above. Then we calculated the extreme values and correlations of the Von Mises stress on the intervertebral discs of the three models under the six states, including forward flexion, backward extension, left bending, right bending, left rotation and right rotation. The activity of the segment and the related comparative study were implemented to determine the impact of articular process resection in different diameters on the biomechanical stability of the lumbar spine.

### ROM-operating segment (L4/5)

**Fig. 4:**
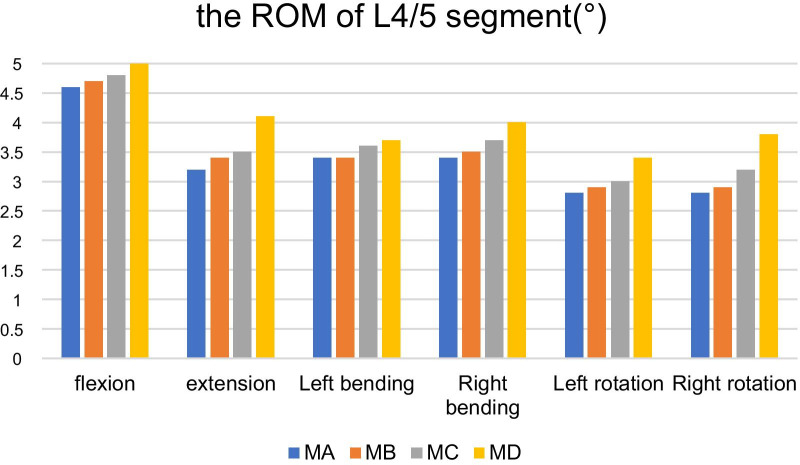
It shows the comparison of the ROM of L4/5 segment in the MA with MB, MC and MD under a torque of 7.5 Nm in forward flexion, backward extension, left bending, right bending, left rotation and right rotation. According to the results obtained by our study, in backward extension, MB and MC increased the ROM of L4/5 segment by 6.25% and 9.38% respectively, while that of MD increased by 28.13%. In right bending, the MB and MC increased the ROM of L4/5 segment by2.94% and 8.82%, respectively, while that of MD increased by 17.65%.In right rotation, the MD notably increased the ROM of L4/5 segment by 35.71%, and this was the most significant increase in L4/5 motion segment among all loading cases

The ROMs of MB, MC and MD are 4.7°, 3.4°, 3.4°, 3.5°, 2.9°, 2.9° (MB) 4.8°, 3.5°, 3.6°, 3.7°, 3.0°, 3.2° (MC) 5.0°, 4.1°, 3.7°, 4.0°, 3.4°, 3.8° (MD) in the conditions of forward flexion, backward extension, left bending,right bending, left rotation and right rotation respectively, increased by 2.17%, 6.25%, 0%, 2.94%, 3.57% and 3.57% (model B), 4.35%, 9.38%, 5.56%, 8.82%, 7.14%, 14.28% (Model C), 8.70%, 28.13%, 8.82%, 17.65%, 21.43%, 35.71% (Model D), compared with the ROM of MA which is shown in Table 3 (Fig. 4).

**Table 3:** The ROM of L4/5 segment after facetectomy in different diameters on L4 inferior articular process (°)

Model	Flexion	Extension	Left bending	Right bending	Left rotation	Right rotation
MA	4.6	3.2	3.4	3.4	2.8	2.8
MB	4.7	3.4	3.4	3.5	2.9	2.9
MC	4.8	3.5	3.6	3.7	3.0	3.2
MD	5.0	4.1	3.7	4.0	3.4	3.8

### ROM-Adjacent segment(L3/4)

**Fig. 5:**
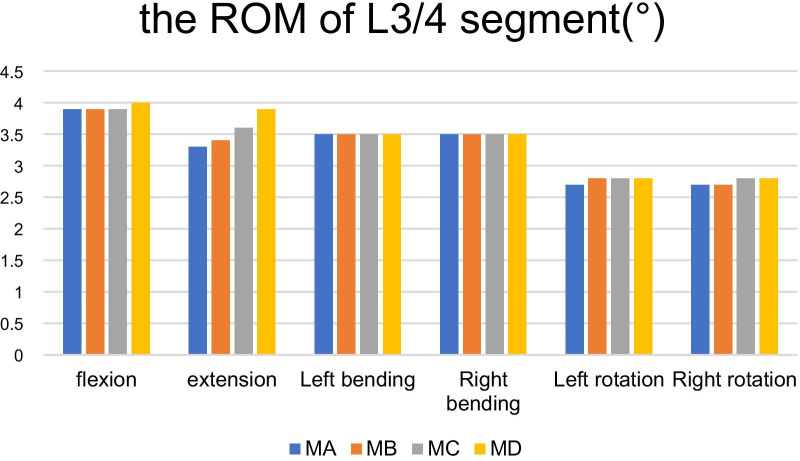
It shows the comparison of the ROM of L3/4 segment in MA with MB, MC and MD under a torque of 7.5 Nm in forward flexion, backward extension, left bending, right bending, left rotation and right rotation. As it is manifested in the figure, neither MB nor MC increased the ROM of L3/4. Even that of the model D increased in the state of backward extension

The ROMs of MB, MC and MD are 3.9°, 3.4°, 3.5°, 3.5°, 2.8°, 2.7° (MB) 3.9°, 3.6°, 3.5°, 3.5°, 2.8°, 2.8° (MC) 4.0°, 3.9°, 3.5°, 3.5°, 2.8°, 2.8° (MD) in the conditions of forward flexion, backward extension, left bending, right bending, left rotation and right rotation respectively, increased by 0%, 3.03%, 0%, 0%, 3.70%, 0% (MB), 0%, 9.10%, 0%, 0%, 3.70%, 3.70% (MC), 2.56%, 18.2%, 0%, 0%, 0%, 3.70%, 3.70% (MD) compared with MA which depicted in Table 4 (Fig. 5).

**Table 4:** The ROM of L3/4 segment after facetectomy in different diameters on L4 inferior articular process (°)

Model	Flexion	Extension	Left bending	Right bending	Left rotation	Right rotation
MA	3.9	3.3	3.5	3.5	2.7	2.7
MB	3.9	3.4	3.5	3.5	2.8	2.7
MC	3.9	3.6	3.5	3.5	2.8	2.8
MD	4.0	3.9	3.5	3.5	2.8	2.8

### Intervertebral disc stress-operating segment (L4/5)

**Fig. 6:**
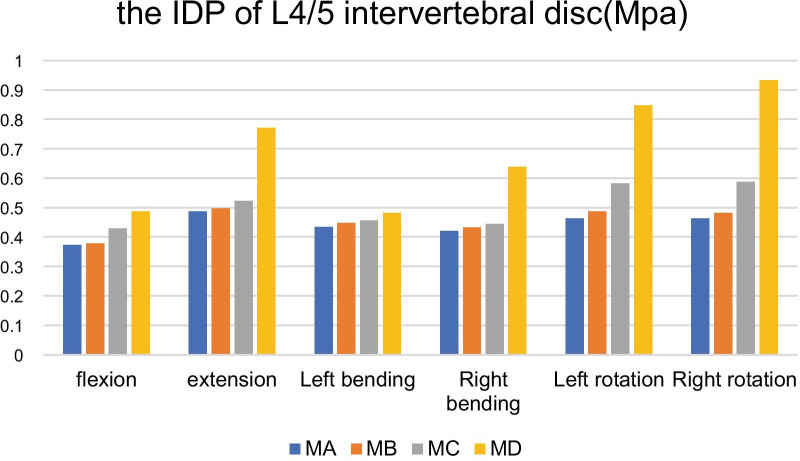
It shows the comparison of the IDP of L4/5 segment in MA with MB, MC and MD under a torque of 7.5 Nm in forward flexion, backward extension, left bending, right bending, left rotation and right rotation. As it depicted, in backward extension, MB and MC increased the IDP of L4/5 by 11.7% while M3 increased. In left rotation, M2 and M3 increased the IDP by 10% and 5.2%, while M2 and M3 increased the IDP of L4/5 by 8.2% and 4.1% in right rotation

Under the condition of forward flexion, backward extension, left and right bending and rotation, the IDP on L4/5 increased by 1.61%, 2.06%, 3.23%, 2.61%, 5.18% and 5.62% (MB), 9.95%, 19.96%, 4.84%, 12.83%, 25.70%, 37.80%(MC). 19.89%, 58.64%, 10.83%, 51.78%, 50.54%, 64.58% (MD) respectively compared with MA which is expressed in Table 5 (Fig. 6).

**Table 5:** The IDP of L4/5 intervertebral disc after facetectomy in different diameters on L4 inferior articular process (MPa)

Model	Flexion	Extension	Left bending	Right bending	Left rotation	Right rotation
MA	0.372	0.486	0.434	0.421	0.463	0.463
MB	0.378	0.496	0.448	0.432	0.487	0.482
MC	0.429	0.523	0.455	0.445	0.582	0.586
MD	0.486	0.771	0.481	0.639	0.847	0.932

### Intervertebral disc stress—adjacent segments (L3/4)

**Fig. 7:**
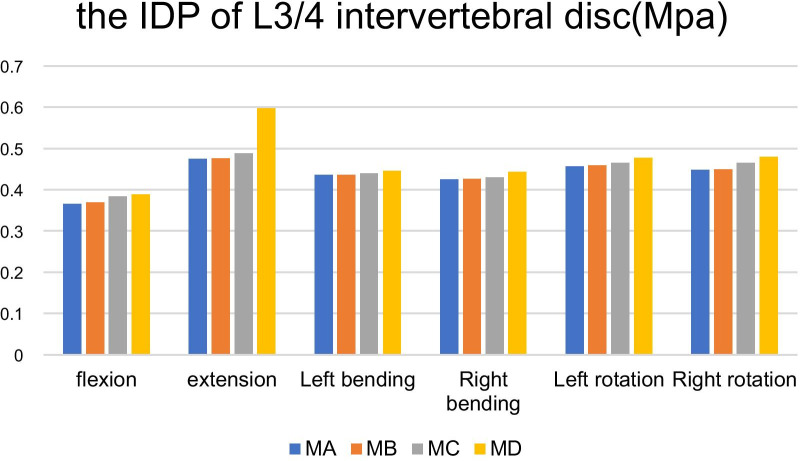
It shows a comparison of the IDP of L3/4 segment in MA with MB, MC and MD under a torque of 7.5 Nm in forward flexion, backward extension, left bending, right bending, left rotation and right rotation. According to the Figure, neither MB nor MC increased the IDP of L3/L4 segment, while MD increased only in backward extension

Compared with MA, under the conditions of forward flexion, backward extension, left bending, right bending, left rotation and right rotation, the IDP on L3/4 of model with facetectomy in a diameter of 7.5 mm, 10 mm and 15 mm increased by 0.82%, 0.21%, 0.23%, 0.47%, 0.44%, 0.45% (MB), 2.47%, 2.95%, 0.92%, 1.18%, 1.97%, 3.80% (MC), 3.29%, 23.84%, 2.30%, 4.48%, 4.61%, 7.16% (MD) respectively as is shown in Table 6 (Fig. 7).

**Table 6:** The IDP of L3/4 intervertebral disc after facetectomy in different diameters on L4 inferior articular process (MPa)

Model	Flexion	Extension	Left bending	Right bending	Left rotation	Right rotation
MA	0.365	0.474	0.435	0.424	0.456	0.447
MB	0.368	0.475	0.436	0.426	0.458	0.449
MC	0.384	0.488	0.439	0.429	0.465	0.464
MD	0.388	0.597	0.445	0.443	0.477	0.479

## Discussion

### Biomechanical study of graded resection of lumbar facet joint

The articular process of the lumbar spine plays an important role in the motion of every single lumbar segment, which forms a "three-joint system" with the intervertebral disc, enabling the lumbar spine to complete compound movement in triaxial and six-directional section. Its effect of anti-compression, anti-shear, anti-rotation and anti-tension could somehow maintain the stability of the lumbar spine [17–20]. In the view of anatomical structure of the lumbar articular process, the inferior facet of the superior vertebral body is located on the posterior medial side of the superior articular process of the inferior vertebral body, and the inferior articular process of the superior vertebral body is surrounded by the superior articular process of the lower vertebral body. The articular surface is at an angle of almost 90°with the transverse section and 45°with the coronal plane. As if the inferior articular process is a tenon and the superior articular process is the sockets. Thus the two parts are firmly locked together to ensure the stability of the lumbar spine [21, 22]. Whichever in lumbar open surgery or endoscopic surgery, in order to reveal the intervertebral disc and complete decompression, it is usually necessary to remove part of the facet joint. However, the excessively large scope of facet joint resection will seriously destroy the mechanical stability of the corresponding segment [23]. Many literature studies [24–26] have shown that if the resection range of lumbar facet joint greater than half of it will make a huge difference in the stability of the lumbar spine. Li et al. [27] performed intervertebral facetectomy by reamers with diameters of 7.5 mm and 10 mm on fresh specimens of spinal functional units (L4/5) of 6 cadavers, and observed its effect on the stability of lumbar vertebrae. The results showed that the intervertebral facetectomy of 10 mm reduced the lateral bending stability of L4/5. The reason probably was, that the ventral part of the L5 superior articular process was partially resected. Meanwhile the tip of superior articular process, part of articular surface and ventral articular capsule were resected as well. The stability of L4/5 is seriously affected. Erbulut [28] simulates 50% resection, 70% resection, total resection of the left facet joint and total resection of bilateral facet joint by three-dimensional finite element method respectively. The results show that with the increase of resection range of the facet joint, the range of motion of L4/5 increases gradually, in especial significantly in the condition of backward extension, left rotation and right rotation. The study further confirmed the importance of lumbar facet joint in preserving lumbar stability.

### Biomechanical effects of different sizes of facetectomy on the operating segment (L4/5)

According to the experimental study, under the conditions of flexion, extension, left and right flexion and rotation, compared with the normal model, the ROM of L4/5 of the three models increased. The above data in the results shows that the effect brought by L4 inferior articular process disectomy on the range of motion of L4/5 increases with the gradual going up of the resection diameter. Especially the MD changed significantly greater than MB and MC. The increase of ROM is the most obvious in the conditions of backward extension, right flexion and right rotation.

During forward flexion and backward extension, the lumbar functional unit is mainly embodied in the by and away between the distance of the inferior articular process of the superior vertebral body and the superior articular process of the lower vertebral body. Adams et al. [29] found that the facet joint is separated and the joint capsule is in tense condition during forward flexion, so it is considered that the resistance against forward flexion is mainly provided by joint ligament rather than the effect of bone structure. The study also indicated that the facet joint is an important structure that limits the range of backward extension of the lumbar motion segment. It shows that when the lumbar spine is in the backward extension state, the superior and inferior articular processes mainly contact each other on the posterolateral side which could produce higher stress, thus limiting the range of backward extension of the lumbar spine. The larger the range of inferior articular process disectomy is, the greater the destruction of articular capsule and articular surface is. As a result, the corresponding segment of lumbar spine loses bony barrier during backward extension, so the range increases. Nevertheless, the limitation of forward flexion mainly depends on the joint capsule and joint capsule ligament which are slightly destroyed during facetectomy, so it makes no difference [30]. As is shown in the study, under backward extension, the ROM of MB is 6.25% higher than that of MA and the ROM of MC is 9.38% higher than that of MA. Moreover, the ROM of MD is 28.13% higher than that of MA under the state. Mentioned above further confirmed that the larger the resection range of the facet joint is, the greater the range of backward extension is.

The effect of unilateral facetectomy on lumbar motion segment indicated that unilateral facetectomy had a great influence on the range of motion of contralateral bending, but had little effect on the range of motion of ipsilateral bending [31]. Li et al. [32] analyzed the contact area and contact stress on the facet joint surface under a single moment load by three-dimensional finite element method. The results showed that there exits a greater contact stress on the anterior medial lower part and the posterolateral upper part of the contralateral facet surface than the stress on the middle and lower part of the ipsilateral facet joint surface. The above results confirm that under the action of pure torque, one side of the facet joint mainly restricts the contralateral bending of the lumbar motion segment, but has little effect on the ipsilateral bending. The facet joints and intervertebral discs of the lumbar vertebrae form a three-joint complex to maintain the stability of the lumbar spine. After facet resection, this mechanical balance is destructed. When bending to ipsilateral side, the fulcrum of the articular process is lost. While bending to the opposite side, the limiting effect of the articular capsule ligament of this side is lost, so the range of lateral bending of this segment will increase [33]. In this study, with the rise in the resection area of the facet joint, the range of motion of L4/5 lateral bending also increase accordingly, particularly in the state of contralateral bending. The experimental results show that in the state of left bending, the ROM of MB, MC and MD increased by 0%, 5.56% and 8.82%, respectively, compared with MA, and increased by 2.94%, 8.82% and 17.65% respectively in the state of right bending, which is basically consistent with the results of previous literature.

The facet joint surface of the lower lumbar vertebra is at an angle of almost 45°toward the sagittal plane, and the joint space is very narrow, causing the bony barrier even when the lumbar spine rotates at a very small range, thus limiting the further rotation of the lumbar spine. The facet joint is the main structure that limits the rotation of the lumbar vertebrae. In the process of rotation, the contact area of the contralateral facet joint increases and against the movement, thus limiting the range of rotation. The ipsilateral two articular processes are separated from each other, and the limitation of the posterolateral articular capsule and articular capsule ligament could further limit the rotational movement of the lumbar spine [34, 35]. In this study, the left inferior articular process of the corresponding lumbar segment was resected and when rotating to the right, the left facet joint lost its bony barrier, so the range of motion increased. Oppositely, when rotating to the left, due to the destruction of the articular capsule ligament, the restriction on left rotation declined, hence the ROM of left rotation also rose, but not as significant as right rotation. The range of facetectomy in MD is quite larger than that of MB and MC, causing greater damage to the articular surface and articular capsule ligament, making more difference to MD in the rotational range of motion of L4/5.

Under the six conditions, the IDP on L4/5 increased, after L4 inferior articular process facetectomy in diameter of 7.5 mm, 10 mm, 15 mm. It could be inferred that the facetectomy of L4 inferior articular process by reamer with different diameters of 7.5 mm, 10 mm and 15 mm has a certain effect on the IDP of L4/5. The IDP of MD is significantly higher than that of the MB and MC increasing by 58.64%, 51.78%, 50.54% and 64.58%, respectively, under the conditions of backward extension, right bending, left rotation and right rotation. The ROM of MD on L4/5 also increased prominently under the conditions of backward extension, right bending and right rotation. Thus, it could indicate that the change of VonMises stress in the corresponding segment of the intervertebral disc is obviously correlated with the change of the corresponding range of motion. In addition to bearing part of the axial load, the facet joints on both sides can resist most of the shear force exerted on the lumbar spine, while the rest are mainly borne by the intervertebral disc. Therefore, bilateral facet joints can protect the intervertebral disc from adverse reactions caused by abnormal stress during bending and rotation. The injury and degeneration of facet joints will lead to the obvious weakening of load-bearing function, and the mechanical center of lumbar load will move forward to the intervertebral disc, and the stress undertook by intervertebral disc will increase [36, 37]. In MD, the whole inferior articular process of L4 was almost removed, losing the bony barrier and the anti-shear&torsion ability as well. Simultaneously, it lost the bony block from the left facet joint during backward extension only remaining the right facet joint undertaking the backward extension stress. Losing the support of the left facet joint during right bending as well as the bony barrier and anti-shear of the left side in the state of left and right rotation, so it will enormously increase the stress of L4/5 intervertebral disc and accelerate intervertebral disc degeneration.

### Biomechanical effects of different sizes of facetectomy on adjacent segments (L3/4)

Compared with the normal model, under the conditions of forward flexion, backward extension, left bending, right bending, left rotation and right rotation, the ROM of MB, MC and MD increased. It indicated that reamers with a diameter of 7.5 mm, 10 mm and 15 mm have no obvious effect on the ROM of L3/4 in forward flexion, left and right flexion and left and right rotation, but have a slight influence only in the state of backward extension particular in the diameter of 15 mm.

Compared with the normal model, under the six conditions, the IDP on L3/4 of model with facetectomy in a diameter of 7.5 mm, 10 mm and 15 mm increased. It could be inferred that the biomechanical effect of three different diameter facetectomy on the IDP of the adjacent segment (L3/4) is apparently less than that of the L4/5. While the L4 inferior articular process resection in a diameter of 7.5 mm and 10 mm has little influence on the L3/4 intervertebral disc stress, but only after the 15 mm-disectomy, especially in the backward extension state, it shows significant change. The results suggested that when the diameter of facetectomy on L4 is greater than a certain value, it may begin to have a corresponding effect on the intervertebral disc stress of the adjacent segments, and this effect may accelerate the degeneration of the adjacent segments.

## Conclusions

To sum up, the normal L3–5 three-dimensional finite element model established in this study has a good geometric configuration, and the imitation of L4 inferior articular process resection in diameter of 7.5 mm, 10 mm and 15 mm could well perform the clinical situation. Conclusion came out that there was a significant correlated change in the VonMises stress and the range of motion of the corresponding segment based on the facetectomy in different sizes. Precisely, the mechanical effect brought by facetectomy in diameter of 15 mm is significantly greater than that brought by facetectomy in diameter of 7.5 mm and 10 mm, and even has a relevant effect on the range of motion and the stress of intervertebral disc of adjacent segments.

### Limitation

This study only reflects the effect of immediate postoperative influence on the stability of the corresponding segments of the lumbar spine. The human body has the ability of compensation and self-repair, and could rehabilitate part of the injured tissue spontaneously after the injury. In this experiment, the midterm and long-term curative effect and stability changes of the operation have not been involved in the research, so further targeted clinical research is needed in the future. The stability of the lumbar spine is affected by various factors. This study only describes the effects brought by bone resection on facet joints included related joint capsule ligament injuries on lumbar stability, without taking into account the factor of muscles and ligaments around the vertebral body. It is surely anticipated that these factors will be embraced in the study one day.

## Abbreviations

ROM: Range of motion
IDP: Intradiscal pressure
FE: Finite element
CT: Computed tomography
MSCT: Multi-sliced spiral computed tomography
MRI: Magnetic resonance imaging
LSS: Lumbar spinal stenosis
LDH: Lumbar disc herniation
PELD: Percutaneous endoscopic lumbar discectomy
PEID: Percutaneous endoscopic interlaminar discectomy
PETD: Percutaneous endoscopic transforaminal disectomy
IAR: Imagery analysis report
NVs: Normalized Von Mises
NRi: Normalized rotation
